# Immune derangement occurs in patients with H7N9 avian influenza

**DOI:** 10.1186/cc13788

**Published:** 2014-03-24

**Authors:** Wei Wu, Yu Shi, Hainv Gao, Weifeng Liang, Jifang Sheng, Lanjuan Li

**Affiliations:** 1 Collaborative Innovation Center for Diagnosis and Treatment of Infectious Diseases, State Key Laboratory of Diagnostic and Treatment of Infectious Diseases, The First Affiliated Hospital, Zhejiang University School of Medicine, Qingchun Road No 79, Hangzhou 310003, China

## Abstract

**Introduction:**

Currently, little is known about the immunological characteristics of patients with avian influenza A (H7N9) virus infection.

**Methods:**

The numbers and percentages of peripheral blood immune cells were measured in 27 patients with laboratory-confirmed H7N9 virus infection and 30 healthy controls (HCs). The functional phenotypes of T cells and monocytes, as well as serum cytokine levels, were analyzed by flow cytometry.

**Results:**

There were 19 patients (70.4%) with acute respiratory distress syndrome, 13 (48.1%) with secondary respiratory infection, 20 (74%) with systemic inflammatory response syndrome (SIRS; defined as having at least two concurrent SIRS components), 18 (66.7%) with lymphocytopenia and 11 (40.7%) with reduced numbers of monocytes. In comparison with levels in the HCs, the levels of serum interleukin 6 (IL-6), IL-8 and IL-10 and the percentages of CD38+ or Tim-3+ T cells were significantly increased. However, the percentages of human leukocyte antigen-DR + and Tim-3+ monocytes were significantly decreased in patients compared with HCs.

**Conclusions:**

Patients with avian H7N9 virus infection display profound SIRS concomitantly with an anti-inflammatory response, which may be associated with the rapid progression of and high mortality associated with this novel viral disease.

## Introduction

Recently in China, an outbreak of influenza occurred that was caused by a novel influenza A (H7N9) viral infection of avian origin. According to published reports, patients with H7N9 virus infection present with rapid, progressive pneumonia commonly leading to the development of acute respiratory distress syndrome (ARDS), respiratory failure and even multiorgan dysfunction syndrome [1]. More importantly, patients with H7N9 virus–mediated influenza have a high mortality rate [2]. Previous studies have revealed the clinical characteristics [1], epidemiology [3-5] and virology [6,7], laboratory diagnosis, and treatment of patients with H7N9 virus infection [8,9]. However, little is known about the impact of H7N9 virus infection on the immune system.

In this paper, we describe the cytokine profiles and functional phenotypes of immunocompetent cells in 27 patients with H7N9 virus–mediated influenza and 30 healthy controls (HCs). We determined the functional phenotypes of immunocompetent cells and serum cytokine profiles of the participants. We describe the cytokine profiles and functional phenotypes of immunocompetent cells in 27 patients.

## Materials and methods

### Patients

We recruited 27 patients with H7N9 virus–mediated influenza and 30 healthy controls (HCs). The patients with avian influenza were recruited from the Inpatient Service at The First Affiliated Hospital of Zhejiang University School of Medicine between 10 and 22 April 2013. Individual patients with H7N9 were diagnosed on the basis of clinical symptoms and laboratory-confirmed H7N9 virus infection. Patients with ARDS were diagnosed according to standard criteria [10]. Thirty age- and gender-matched HCs were recruited at the Physical Examination Center of the hospital during the same period. The mean age of the HCs was 57 ± 12 years, and 56.7% of them were male (17 participants). The exclusion criteria were (1) coinfection with hepatitis B virus (HBV), hepatitis C virus (HCV) or HIV; (2) the presence of a common autoimmune disease and tumor; and/or (3) a recent history of chemotherapy, radiotherapy or use of immunosuppressants. Written informed consent was obtained from each participant. The experimental protocol was established in accordance with the Declaration of Helsinki and approved by the Ethics Committee of The First Affiliated Hospital of Zhejiang University School of Medicine. The patients’ demographic and clinical characteristics are shown in Table 1.

**Table 1:** **Demographic, epidemiologic and clinical characteristics of 27 patients with H7N9 virus infection**^**a**^

**Variables**	**Values**
Age (years)	62 ± 14
Male, *n* (%)	16 (59.3)
Smoker, *n* (%)	8 (29.6)
Exposure to live poultry, *n* (%)	16 (59.3)
Underlying conditions, *n* (%)	
Any	15 (55.6)
Hypertension	13 (48.1)
Coronary heart disease	2 (7.4)
Diabetes mellitus	3 (11.1)
Chronic obstructive pulmonary disease	1 (3.7)
Other	7 (25.9)
Days between disease onset and admission	1.0 (0.5 to 19.0)
White blood cell count (10^9^/L)	5.1 ± 3.0
Hemoglobin (g/dl)	123 ± 24
Platelet count (10^9^/L)	132 ± 46
Alanine aminotransferase (U/L) (*n* = 26)	36 (12 to 262)
Creatinine (μmol/L)	57 (25 to 266)
Potassium (mmol/L)	4.37 ± 0.48
Sodium (mmol/L)	139 ± 4
International normalized ratio	1.08 ± 0.093
D-dimer (μg/L) (*n* = 26)	2,790 (120 to 42,920)
PaO_2_:FiO_2_ ratio	143.26 ± 58.38
Chest radiological abnormalities	
Involvement of both lungs (*n* = 26)	18 (69.2)
Ground-glass opacities (*n* = 21)	2 (8.7)
Pulmonary consolidation	25 (92.6)
APACHE II score	22.26 ± 7.70
Complications	
Acute respiratory distress syndrome, *n* (%)	19 (70.4)
Secondary infection, *n* (%)	13 (48.1)
Liver damage, *n* (%) (*n* = 26)	12 (44.4)
Shock, *n* (%)	6 (22.2)
Acute renal injury, *n* (%)	1 (3.7)
Antiviral therapy with oseltamivir, *n* (%)	27 (100)
Glucocorticoid therapy, *n* (%)	10 (37.0)
Mechanical ventilation, *n* (%)	13 (48.1)
Extracorporeal membrane oxygenation, *n* (%)	9 (33.3)

^**a**^**APACHE II**, Acute Physiology and Chronic Health Evaluation II; **PaO**_2_: FiO_2_, ratio of partial pressure of arterial oxygen to fraction of inspired oxygen.

Continuous data were expressed as mean ± SD or median (range), and categorical data were represented as number (percentage).

### Laboratory examination of H7N9 virus patients

Sputum samples were collected from individual patients immediately after hospitalization, and the presence of H7N9 virus in the collected sputum samples was determined by real-time RT-PCR as previously described [8]. Briefly, the presence of the M, H7 and N9 genes of the H7N9 virus was detected by TaqMan real-time RT-PCR assays (Applied Biosystems, Foster City, CA, USA) using specific primers. The sequences of primers and probes used were as follows: M forward: 5′-GAGTGGCTAAAGACAAGACCAATC-3′), M reverse: 5′-TTGGACAAAGCGTCTACGC-3′ and M probe: 6-carboxyfluorescein (6-FAM)-TCACCGTGCCCAGTGAGCGAG-black hole quencher 1 (BHQ1); H7 forward: AGAGTCATTRCARAATAGAATACAGAT, H7 reverse: CACYGCATGTTTCCATTCTT and H7 probe: 6-FAM-AAACATGATGCCCCGAAGCTAAAC-BHQ1; and N9 forward: GTTCTATGCTCTCAGCCAAGG, N9 reverse: CTTGACCACCCAATGCATTC and N9 probe: hexachlorofluorescein-TAAGCTRGCCACTATCATCACCRCC-BHQ1. The sensitivity of these RT-PCR assays was approximately 100 copies/ml RNA.

### Flow cytometry

Venous blood samples were collected from individual patients immediately after hospitalization and from HCs when they visited the hospital. To characterize the frequency of T, natural killer (NK) and B cells, individual blood samples (0.5 ml) were stained with the following antibodies: phycoerythrin (PE) cyanine 5–conjugated (Pcy5) anti-CD3, fluorescein isothiocyanate (FITC)-conjugated anti-CD4, PE-conjugated anti-CD8 (BD Biosciences, San Jose, CA, USA), PE-conjugated anti-CD16/anti-CD56 or FITC-conjugated anti-CD19 (Beckman Coulter, Brea, CA, USA). Furthermore, to characterize T-cell immunoglobulin mucin 3–positive (Tim-3+) or CD38+ T cells, blood samples were stained with the following antibodies: Pcy5-conjugated anti-CD3, FITC-conjugated anti-CD4, FITC-conjugated anti-CD8, PE-conjugated anti-Tim-3 (R&D Systems, Minneapolis, MN, USA) or PE-conjugated anti-CD38 (Beckman Coulter). In addition, to characterize Tim-3+ and HLA-DR + monocytes, whole-blood samples were stained with antibodies against FITC-conjugated anti-CD14 and allophycocyanin-conjugated anti-Tim-3 (R&D Systems) or PE antibodies against major histocompatibility complex class II cell surface receptor encoded by the human leukocyte antigen (anti-HLA-DR) (BD Biosciences). The isotype-matched immunoglobulins were used as controls. The frequency of different types of immunocompetent cells was characterized by flow cytometry, and at least 1 × 10^5^ events were analyzed using an FC500 MPL flow cytometer (Beckman Coulter) or an Accuri C6 cytometer (BD Biosciences).

### Measurement of serum cytokines

Individual serum samples were obtained from patients immediately after hospitalization and from HCs when they visited the hospital. The concentrations of serum interferon γ (IFN-γ), tumor necrosis factor α (TNF-α), TNF-β, interleukin 1β (IL-1β), IL-2, IL-4, IL-5, IL-6, IL-8, IL-10 and IL-12P70 in individual participants were determined by cytometric bead array (CBA) using a FlowCytomix Simplex Kit (Bender MedSystems/eBioscience, San Diego, CA, USA), according to the manufacturer’s instructions, which were described previously [11]. The concentrations of individual serum cytokines were determined using standard curves established with the individual recombinant cytokines provided. The limitation of detection was 1.6 pg/ml for IFN-γ, 3.2 pg/ml for TNF-α, 2.4 pg/ml for TNF-β, 4.2 pg/ml for IL-1β, 16.4 pg/ml for IL-2, 20.8 pg/ml for IL-4, 1.6 pg/ml for IL-5, 1.2 pg/ml for IL-6, 0.5 pg/ml for IL-8, 1.9 pg/ml for IL-10 and 1.5 pg/ml for IL-12P70.

### Statistical analysis

Continuous data are expressed as mean ± SD or median (range), and categorical data are given as percentages. Comparison of the data between the two groups was analyzed by Student’s *t*-test, Mann–Whitney *U* nonparametric test and χ^2^ test using SPSS 16.0 for Windows software (SPSS, Chicago, IL, USA). A two-sided *P*-value less than 0.05 was considered statistically significant.

## Results

### Demographic, epidemiological and clinical characteristics of the study population

A total of 27 patients with confirmed avian-origin influenza A (H7N9) virus infection and 30 HCs were recruited for participation in this study. Their demographic and clinical characteristics are presented in Table 1. There were no significant differences between the patients and HCs in our sample population with regard to age or gender. After hospital admission, sputum samples were collected from individual patients and subjected to characterization of H7N9 virus genes. The sputum samples from all patients were positive for the M, H7 and N9 genes as determined by RT-PCR, confirming that all the patients had H7N9 avian-origin influenza virus infection. Of the 27 patients, 8 (29.6%) were smokers and 16 (59.3%) had a definitive history of poultry exposure. Furthermore, 55.6% of the patients had preexisting chronic diseases, such as hypertension.

Chest radiographs showed that all patients displayed significant changes, and 92.6% of them had bilateral consolidation in the lungs. Most patients were in critical condition, with an average Acute Physiology and Chronic Health Evaluation II score of 22.26 ± 7.70, 70.4% developed ARDS and 48.1% had secondary infections in the respiratory tract. In addition, many patients developed severe complications, including liver damage (44.4%), renal injury (3.7%) and shock (22.2%). All patients were given oral antiviral therapy with oseltamivir, and 10 (37.0%) received glucocorticoid treatment. Approximately one-half of the patients (13 (48.1%) of 27) required mechanical ventilation, and 9 of them received extracorporeal membrane oxygenation.

### Patients with H7N9 avian influenza developed systemic inflammatory response syndrome at admission

Many patients with clinical presentation of H7N9 avian influenza developed systemic inflammatory response syndrome (SIRS)–related clinical symptoms and signs, such as abnormalities in body temperature, heart rate, respiratory rate and leukocyte count [12], suggesting that a hyperactivated inflammatory response may play a pivotal role in disease progression. We found that 70% patients had at least two concurrent SIRS components. In addition, 92.6% of patients had increased levels of serum C-reactive protein, 33.3% had elevated levels of serum procalcitonin and 77.8% had abnormally high erythrocyte sedimentation rates (Table 2).

**Table 2:** **Systemic inflammatory response syndrome components and clinical inflammatory markers in 27 patients with H7N9 virus infection**^**a**^

**Variables**	**Frequency**
Body temperature <36°C or >38°C, *n* (%)	24 (88.9)
Heart rate >90 beats/min, *n* (%)	7 (25.9)
Respiratory rate >20 breaths/min or PaCO_2_ < 32 mmHg, *n* (%)	13 (48.1)
WBC count <4 × 10^9^/L or >12 × 10^9^/L, *n* (%)	13 (48.1)
Maximum two concurrent components, *n* (%)	11 (40.7)
Maximum three concurrent components, *n* (%)	7 (25.9)
Maximum four concurrent components, *n* (%)	2 (7.4)
C-reactive protein >10 mg/dl, *n* (%)	25 (92.6)
Erythrocyte sedimentation rate >20 mm/h (*n* = 18)	14 (77.8)
Procalcitonin >0.5 ng/ml, *n*/*N* (%) (*N* = 24)	8 (33.3)

^a^PaCO_2_, partial pressure of arterial carbon dioxide; WBC, white blood cell.

Data were represented as number (percentage).

### Significantly higher levels of serum cytokines in patients with H7N9 avian influenza

**Figure 1:**
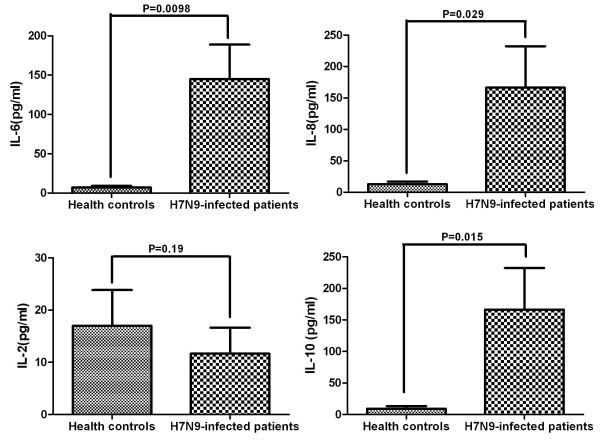
**Levels of circulating cytokines in patients with H7N9 avian influenza.** Serum samples were prepared from 10 patients and 10 healthy controls. We carried out cytometric bead arrays to measure the concentrations of serum interleukin 1β (IL-1β), IL-2, IL-4, IL-5, IL-6, IL-8, IL-10, IL-12p70, tumor necrosis factor α (TNF-α), TNF-β and interferon γ (IFN-γ) in individual samples. Data shown are the mean values of individual cytokines in each participant from two separate experiments, which we analyzed by Mann–Whitney *U* test. Under our experimental conditions, we found no detectable levels of serum IL-1β, IL-4, IL-5, IL-12p70, TNF-α, TNF-β or IFN-γ in these participants (data not shown). Error bars were shown in each column.

SIRS is a consequence of cytokine storm, so we evaluated the levels of serum cytokines by CBA in HCs and in the patients with H7N9 influenza at the time of admission. We detected IL-2, IL-6, IL-8 and IL-10, but we did not detect IFN-γ, TNF-α, TNF-β, IL-1β, IL-4, IL-5 or IL-12p70. We found that the levels of serum IL-6 (*P* = 0.0098), IL-8 (*P* = 0.0010) and IL-10 (*P* = 0.015) in the patients were significantly higher than those in the HCs (Figure 1). However, there was no significant difference in the levels of serum IL-2 between these two groups (*P* = 0.19).

### Alteration in number of peripheral blood immunocompetent cells in patients with H7N9 avian influenza

Next, we examined the numbers of peripheral blood immunocompetent cells. We found that 66.7% of patients developed lymphocytopenia (lymphocytes <0.8 × 10^9^/L) and 40.7% had abnormally low monocyte counts (monocytes <0.12 × 10^9^/L). However, only 25.9% of patients had neutropenia (neutrophils <2.0 × 10^9^/L). Further flow cytometric analysis indicated that the percentages of CD3+ T cells (58.88 ± 16.34% vs. 67.22 ± 9.22%; *P* = 0.020) and CD8+ T cells (20.18 ± 8.58% vs. 24.19 ± 6.06%; *P* = 0.045) were significantly lower in the patients than in the HCs. In addition, we found no significant differences in the frequency of peripheral blood CD3 + CD4+ T cells and CD3 - CD56+ NK cells between the patients and the HCs (37.46 ± 13.50% vs. 38.61 ± 8.84%; P = 0.70 for CD4+ T cells; and 15.10 ± 11.00% vs. 19.73 ± 9.55%; *P* = 0.095 for NK cells) (Table 3).

**Table 3:** **Comparison of counts and percentages of lymphocytes and lymphocyte subsets between patients with H7N9 virus infection and healthy controls**^**a**^

**Variables**	**Patients (*n*** **= 27)**	**Healthy controls (*n*** **= 30)**	***P*-value**
Neutrophils <2.0 × 10^9^/L, *n* (%)	7 (25.9)	None	–
Monocytes <0.12 × 10^9^/L, *n* (%)	10 (40.7)	None	–
Lymphocytes <0.8 × 10^9^/L, *n* (%)	18 (66.7)	None	–
CD3+ T cells (%)	58.88 ± 16.34	67.22 ± 9.22	0.020
CD4+ T cells (%)	37.46 ± 13.50	38.61 ± 8.84	0.70
CD8+ T cells (%)	20.18 ± 8.58	24.19 ± 6.06	0.045
Natural killer cells (%)	15.10 ± 11.00	19.73 ± 9.55	0.095

^a^Statistical analysis was performed by Student’s *t*-test.

Continuous data were expressed as mean ± SD and categorical data were represented as number (percentage).

**Figure 2:**
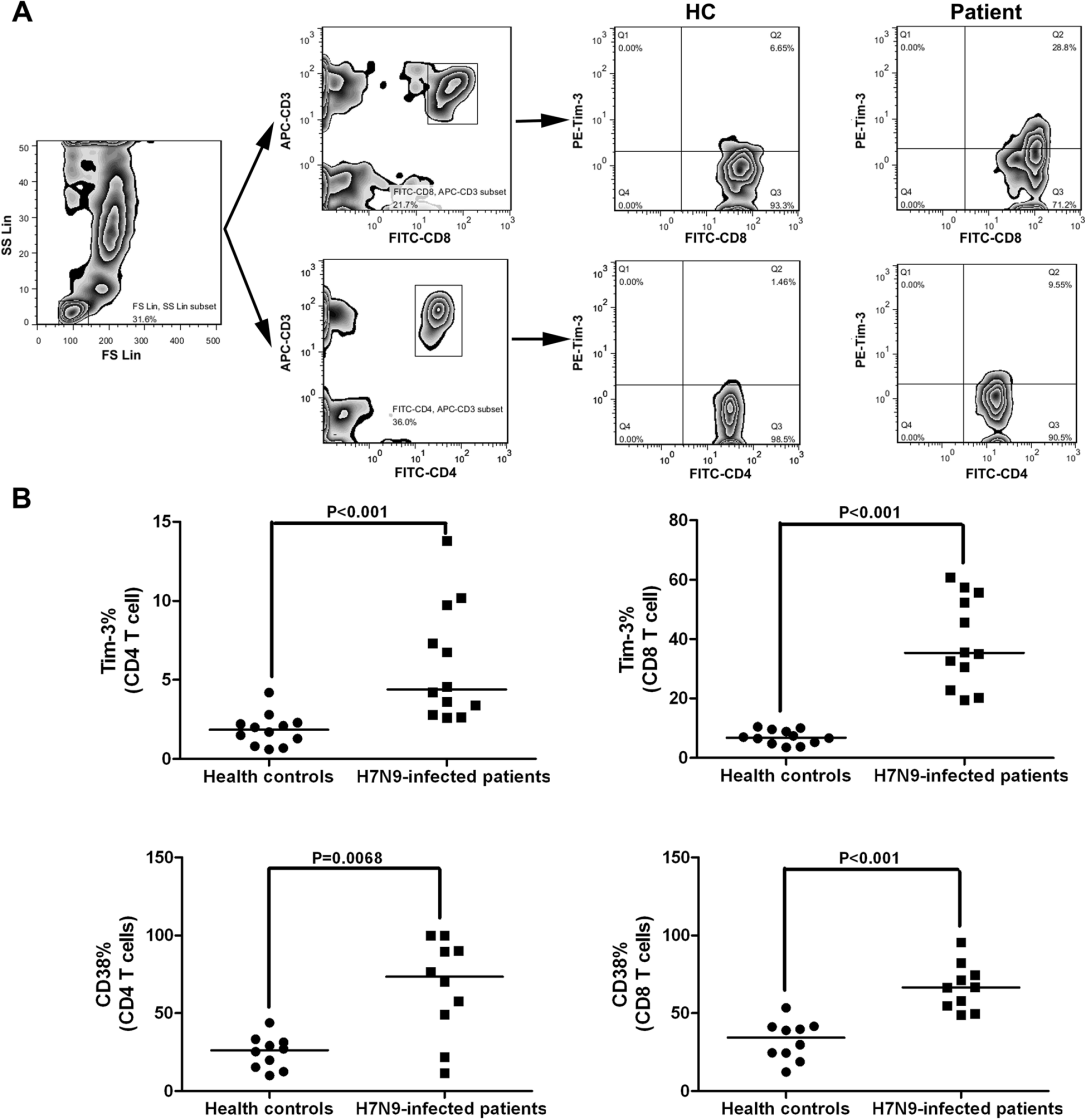
**Flow cytometric analysis of the frequency of CD38+ and Tim-3+ T cells.** Peripheral whole blood was collected from 18 patients and 12 healthy controls (HC). Blood samples were stained with the fluorescent antibodies anti-CD3, anti-CD4/CD8 and anti-CD38 or anti-Tim-3 and were measured for the percentages of CD38+ and Tim-3+ T cells. Data are presented as flowcharts and expressed as the means of individual participants. The Mann–Whitney *U* test was used to analyze the differences between groups. **(A)** Representative flowcharts. FSC Lin, forward scatter, linear scale; SSC Lin, side scatter, linear scale. **(B)** Quantitative analysis of the percentages of CD38+ and Tim-3+ T cells. The horizontal lines indicate the median values for each group. FITC, fluorescein isothiocyanate; Tim-3, T-cell immunoglobulin mucin 3.

CD38 is a marker of T-cell activation, and Tim-3 is a surface marker of T-cell exhaustion [13]. Further characterization of functional T cells revealed that the percentages of peripheral blood CD38 + CD4+ and CD38 + CD8+ T cells in the patients were significantly higher than those in the HCs (*P* = 0.0068 and *P* < 0.001, respectively) (Figure 2). Concomitantly, the percentages of peripheral blood Tim-3 + CD4+ and Tim-3 + CD8+ T cells in the patients were significantly higher than those in the HCs (*P* < 0.001 for both).

**Figure 3:**
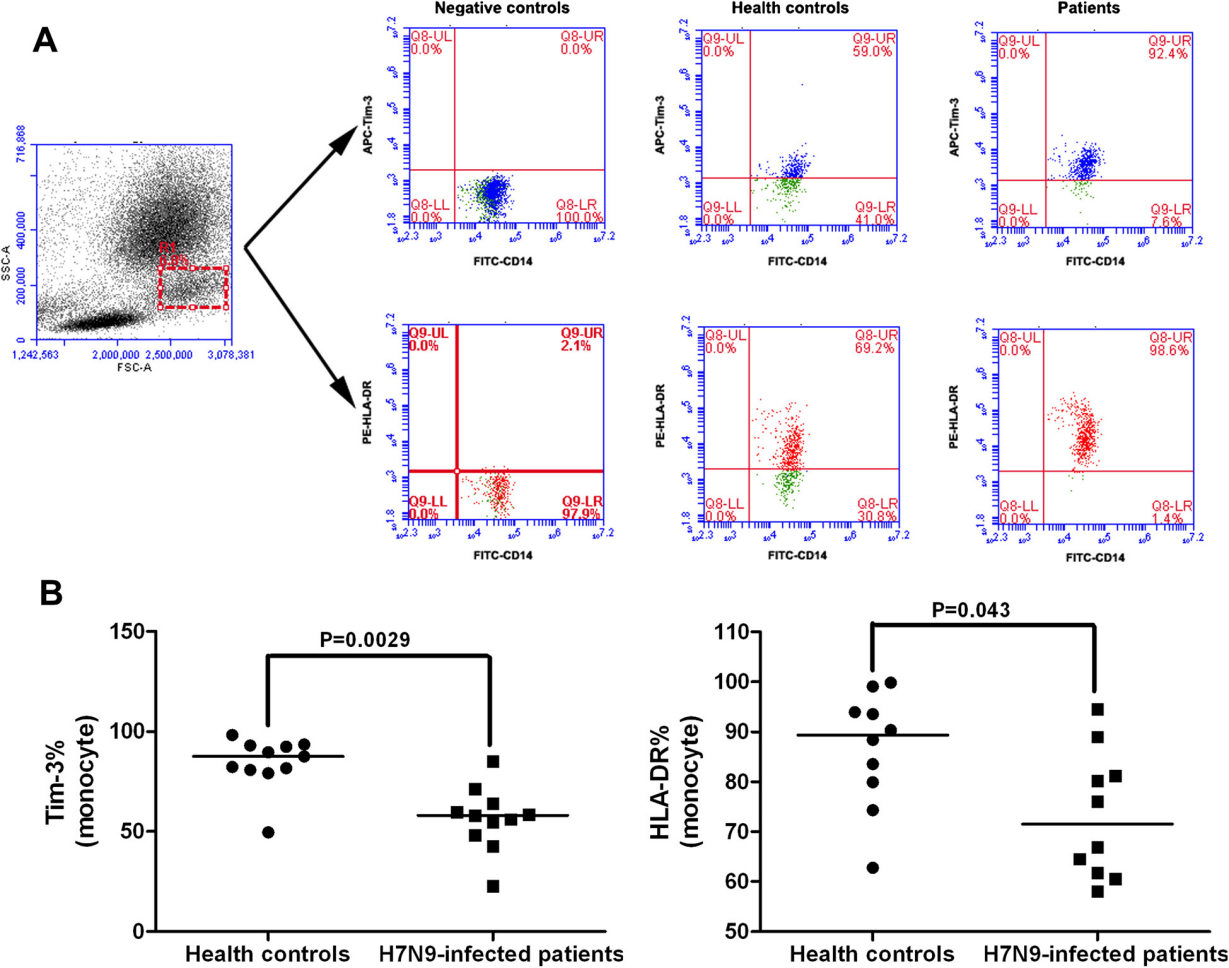
**Flow cytometric analysis of the frequency of HLA-DR + and Tim-3+ monocytes.** Peripheral whole-blood samples were collected from 13 patients and 11 healthy controls, stained with the fluorescent antibodies against CD14 (anti-CD14) and major histocompatibility complex class II cell surface receptor encoded by the human leukocyte antigen (anti-HLA-DR) or T-cell immunoglobulin mucin 3 (anti-Tim-3), and measured for the percentage of HLA-DR + CD14+ or Tim-3 + CD14+ monocytes in the total CD14+ monocytes. Data are presented as flowcharts and expressed as the means of individual participants. The Mann–Whitney *U* test was used to analyze the differences between groups. **(A)** The gating strategy and representative flow cytometric plots of Tim-3+ and HLA-DR + monocytes are shown. FITC, fluorescein isothiocyanate; FSC Lin, forward scatter area; SSC Lin, side scatter area. **(B)** Quantitative analysis.

We next assessed the functional phenotypes of peripheral blood monocytes and found that the percentages of HLA-DR + CD14+ and Tim-3 + CD14+ monocytes in total CD14+ cells in patients were significantly lower than those in the HCs (*P* = 0.043 and *P* = 0.0029, respectively) (Figure 3). Collectively, these data suggest that patients with H7N9 avian influenza had immunodysfunction.

## Discussion

In the present study, we examined the immune system alterations in patients with H7N9 avian influenza. We found that a high percentage of patients developed SIRS accompanied by a high percentage of activated T cells and increased levels of serum cytokines. Concomitantly, many patients displayed lymphocytopenia, abnormally low monocyte counts, T-cell exhaustion and monocyte dysfunction, which are characteristic of immune paralysis.

The presence of SIRS is predictive of organ dysfunction and mortality [14,15]. We found that 70% of patients had at least two concurrent SIRS components and detected significantly higher levels of serum IL-6 and IL-8 in the patients than in HCs, which may explain the high morbidity and mortality associated with this disease. However, we did not detect significant alterations in the levels of serum IFN-γ or TNF-α in these patients. This cytokine profile is analogous to that of patients with severe acute respiratory syndrome (SARS) [16,17]. Furthermore, it has been reported that patients with either H7N9 virus influenza or SARS coronavirus-related illness shared striking similarities with regard to their clinical presentation and disease progression. Therefore, it is possible that the pathogenesis of H7N9 virus infection is similar to that of SARS coronavirus-related infection [18]. Although patients with severe influenza induced by the H1N1 and H5N1 viruses develop a “cytokine storm,” including high levels of serum IFN-γ and TNF-α, which are commonly associated with rapid progression and poor prognosis [11,19,20], we did not detect abnormal levels of serum IFN-γ or TNF-α in patients with H7N9 virus infection. These findings suggest that different immune responses may occur in patients with varying types of influenza virus infection. The investigators in one recent study reported that a high frequency of programmed death receptor 1 (PD-1), and its ligand 1 (PD-L1), that expressed T cells impaired T-cell responses to H1N1 infection in patients with influenza [21]. It is possible that similar mechanisms may underlie the failure to detect abnormal levels of serum IFN-γ and TNF-α in patients with H7N9 virus infection. Although proinflammatory IFN-γ and TNF-α responses usually occur at early stages of immune responses, it is also possible that the failure to detect abnormal levels of serum IFN-γ or TNF-α may stem from missing the very early time point in our study. Therefore, further studies are needed to clarify the mechanisms underlying the pathogenesis of H7N9 virus infection and host immune responses.

Our results show that many patients with severe avian H7N9 influenza developed T-cell lymphocytopenia. Such a phenomenon is commonly reported in patients with SARS. However, pneumonia caused by other common respiratory viruses are usually associated with a normal or elevated lymphocyte count [22]. The lymphocytopenia in patients with H7N9 avian influenza may be a key factor leading to high morbidity and mortality, because lymphocytopenia is an independent risk factor for ARDS, which is a very dangerous condition for patients with secondary infections [1]. The lymphocytopenia in these patients likely derives from the migration of T lymphocytes into the target tissues, such as the lungs. Alternatively, the lymphocytopenia may stem from virus-stimulated, activation-induced T-cell apoptosis and virus infection–related bone marrow suppression [23].

T lymphocytes play a pivotal role in the defense of viral infection by directly killing virus-infected cells. In this study, we found significantly higher frequencies of CD38 + CD4+, CD38 + CD8+, Tim-3 + CD4+ and Tim-3 + CD8+ T cells in patients with H7N9 avian influenza compared with those in the HCs. It is well-known that CD38 and Tim-3 expression are associated with T-cell activation [13]. The higher frequency of CD38+ and Tim-3+ T cells in patients with H7N9 avian influenza indicated that viral infection induced significant T-cell activation. However, Tim-3 on activated T cells usually provides a negative signal for effector T-cell function and leads to T-cell functional exhaustion [13,24,25]. Furthermore, engagement of Tim-3 by its specific ligand of galectin-9 can trigger T-cell apoptosis [26]. Notably, IFN-γ is a potent inducer of galectin-9 protein expression, thus the low IFN-γ expression in patients with severe H7N9 influenza may limit the production of galectin-9, leading to a high frequency of impotent Tim-3+ T cells. Thus, in turn, aberration of T-cell activation in patients with H7N9 avian influenza may render T-cell exhaustion and apoptosis or impotence, leading to poor immune responses. We are interested in further investigating the levels of galectin-9 and T-cell function to discern the precise mechanisms underlying immune responses to H7N9 virus infection in humans.

Monocytes are an important component of the innate immune system, which recognizes pathogens, secretes proinflammatory cytokines and chemokines to initiate the immune response, and presents antigens to trigger adaptive immune responses [27]. In this study, we found a significantly reduced frequency of peripheral blood HLA-DR + CD14+ and Tim-3 + CD14+ monocytes in patients with H7N9 avian influenza, at levels similar to those in patients with septic shock [28], trauma [29] and acute liver failure [30], as well as postoperative patients [31]. In these clinical settings, downregulation of HLA-DR expression usually represents the functional impairment of monocytes and is associated with adverse outcomes. In addition, the reduced levels of Tim-3 expression on monocytes may contribute to the functional deactivation of monocytes, as it was reported previously that constitutive Tim-3 expression in naïve and resting immunocompetent cells promotes inflammation [32].

In addition, we noted that nearly one-half of the patients in our study had evidence of secondary infection, which is very dangerous because secondary bacterial or fungal infection is a common factor leading to mortality in patients with H7N9 avian influenza [1]. We speculate that the increased predisposition to secondary infection of patients is related to the deranged immune response. Therefore, it is crucial for clinicians to pay special attention to patients with severe influenza by modulating immunocompetent cell function to limit adverse consequences in the clinic.

We recognize that our study has limitations, including its small sample size, measurement at one time point for a few markers of functional immunocompetent cells and the lack of antigen-specific T-cell immunity. Therefore, further studies are warranted to measure longitudinally the dynamic changes in the immunological status of patients during the whole time course of H7N9 infection and to assess the function of different types of immunocompetent cells using other functional markers (for example, CD25, CD69, PD-1 and LAG-3) and antigen-specific T-cell immunity by *ex vivo* experiments in a larger sample population. We are interested in further investigating the molecular mechanisms underlying the immunoderangement in patients with H7N9 avian influenza.

## Conclusions

Overall, our findings reveal that patients with H7N9 avian influenza commonly develop SIRS accompanied by T-cell lymphocytopenia and exhaustion, as well as monocyte dysfunction. The immunoderangement may be associated with the high mortality rate associated with this disease.

## Key messages

• Patients with H7N9 avian influenza usually develop ARDS and secondary infection and have a high mortality rate.

• A hyperactivated inflammatory response and an anti-inflammatory response occur concomitantly in patients with H7N9 avian influenza.

• Such immune derangement may contribute to the rapid progression of and high mortality associated with this disease.

## Abbreviations

ARDS: Acute respiratory distress syndrome; CBA: Cytometric bead array; CRP: C-reactive protein; ECMO: Extracorporeal membrane oxygenation; ESR: Erythrocyte sedimentation rate; H7N9: Avian influenza A; HBV: Hepatitis B virus; HCV: Hepatitis C virus; SARS: Severe acute respiratory syndrome; SIRS: Systemic inflammatory response syndrome.

## Competing interests

The authors declare that they have no competing interests.

## Authors’ contributions

WW carried out the RT-PCR and flow cytometric analysis, participated in the design of the study and helped to draft the manuscript. YS carried out the cytokine measurements, participated in the design of the study and helped to draft the manuscript. HG analyzed and interpreted the results and revised the manuscript. WL and JS participated in the design of the study and revised the manuscript. LL conceived of the study, participated in its design and coordination and helped to draft the manuscript. All authors read and approved the final manuscript.
